# Comparison of eukaryotic phylogenetic profiling approaches using species tree aware methods

**DOI:** 10.1186/1471-2105-10-383

**Published:** 2009-11-24

**Authors:** Valentín Ruano-Rubio, Olivier Poch, Julie D Thompson

**Affiliations:** 1 Laboratoire de Biologie et Génomique Intégrative, Département de Biologie et Génomique Structurales, Institut de Génétique et de Biologie Moléculaire et Cellulaire, CNRS/INSERM/UDS, Illkirch, France

## Abstract

**Background:**

Phylogenetic profiling encompasses an important set of methodologies for *in silico *high throughput inference of functional relationships between genes. The simplest profiles represent the distribution of gene presence-absence in a set of species as a sequence of 0's and 1's, and it is assumed that functionally related genes will have more similar profiles. The methodology has been successfully used in numerous studies of prokaryotic genomes, although its application in eukaryotes appears problematic, with reported low accuracy due to the complex genomic organization within this domain of life. Recently some groups have proposed an alternative approach based on the correlation of homologous gene group sizes, taking into account all potentially informative genetic events leading to a change in group size, regardless of whether they result in a *de novo *group gain or total gene group loss.

**Results:**

We have compared the performance of classical presence-absence and group size based approaches using a large, diverse set of eukaryotic species. In contrast to most previous comparisons in Eukarya, we take into account the species phylogeny. We also compare the approaches using two different group categories, based on orthology and on domain-sharing. Our results confirm a limited overall performance of phylogenetic profiling in eukaryotes. Although group size based approaches initially showed an increase in performance for the domain-sharing based groups, this seems to be an overestimation due to a simplistic negative control dataset and the choice of null hypothesis rejection criteria.

**Conclusion:**

Presence-absence profiling represents a more accurate classifier of related versus non-related profile pairs, when the profiles under consideration have enough information content. Group size based approaches provide a complementary means of detecting domain or family level co-evolution between groups that may be elusive to presence-absence profiling. Moreover positive correlation between co-evolution scores and functional links imply that these methods could be used to estimate functional distances between gene groups and to cluster them based on their functional relatedness. This study should have important implications for the future development and application of phylogenetic profiling methods, not only in eukaryotic, but also in prokaryotic datasets.

## Background

**Figure 1:**
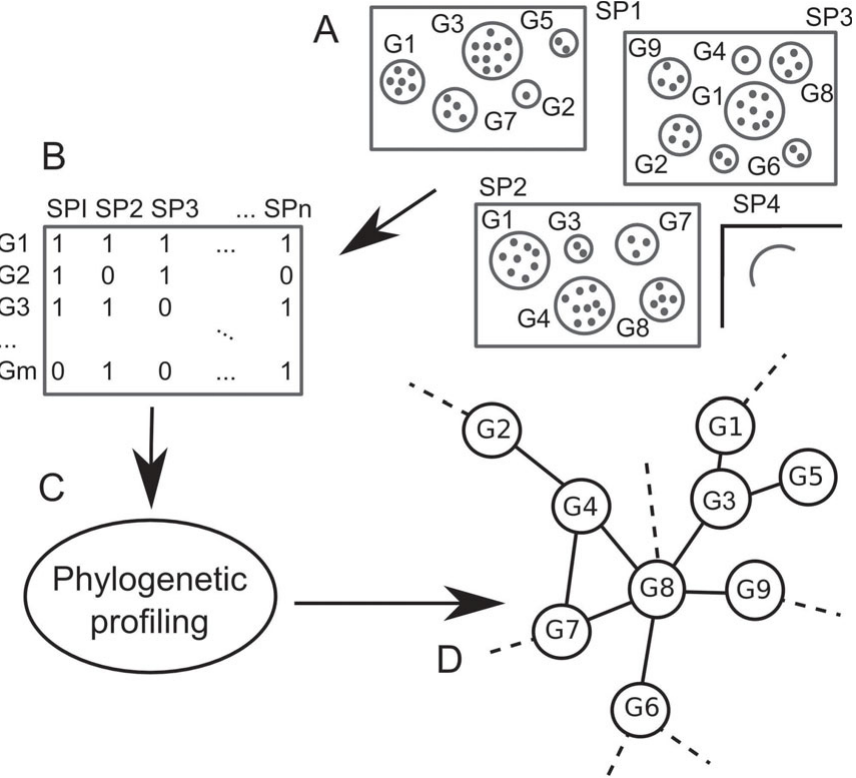
**Schema of a simple phylogenetic profiling pipeline**. A) genes (dots) are grouped using a biologically relevant criterion (e.g. orthology) in a diverse set of genomes (rectangles). B) a matrix is constructed with rows corresponding to groups and columns corresponding to genomes. Each cell in the matrix is assigned a value of 1 (presence) or 0 (absence) of the group in the genome. C) this matrix is used as input to a phylogenetic profiling method that results in a list of potentially related gene groups. D) the output can then be used to build a functional network that may well lead to or give support to further biological findings.

A decade has passed since the introduction of phylogenetic profiles to elucidate functional relationships between gene products. The basic assumption in the phylogenetic profiling approach is that convergent evolution of traits, e.g. gene co-presence and co-absence patterns across genomes are due to inter-dependence between those traits [1]. For example, if the activity of a gene *A *is dependent on the existence of another gene *B*, a fixed deletion of *B *renders *A *redundant and consequently *A *will be eventually lost due to genetic drift as it is no longer subject to selective constraints. Phylogenetic profiles of gene presence-absence are typically encoded as a binary sequence of 1's (presence) and 0's (absence) where each position in the profile corresponds to a particular species under study (Figure 1). Functional dependencies between genes should then result in a smaller number of differences in the corresponding profiles than expected by chance.

Since their introduction, phylogenetic profiling approaches have evolved and a number of novel methods have been developed to quantify the similarity between two profiles or to statistically evaluate the reliability of the predicted functional relationships. These methods can be coarsely divided into two categories depending on whether they take into account the underlying species phylogeny: non-phylogenetic approaches that include pairwise distance comparisons [1], mutual information [2] or Pearson correlation [3] and phylogeny-aware approaches that rely on parsimony [4], maximum-likelihood [5] or kernel-methods [6] based scoring. In theory, phylogeny-aware approaches are preferable due to the statistical bias introduced by gene content inter-dependence between closely related species, and it has been shown that such methods can improve the prediction of functional links in eukaryotic genomes [5]. Nevertheless, these methods remain computationally complex. As a consequence, some groups have proposed phylogeny-based heuristic corrections with the goal of reducing the inherent biases in a computationally efficient manner (e.g. [7]).

Despite the successful application of phylogenetic profiles in a number of studies involving prokaryotic genomes (reviewed in [8]), profiling in eukaryotes has received little attention. Moreover, based on combined prokaryotic and eukaryotic studies, some groups have concluded that the statistical signal is severely disrupted by inclusion of eukaryotic genomes [9,10]. To measure the extent to which these methods can be applied in studies of eukaryotes, Singh and Wall [11] constructed phylogenetic profiles consisting exclusively of eukaryotic genomes and detected some correlation for proteins within the same functional module determined using the Gene Ontology [12]. This finding indicates the presence of at least some statistical signal in eukaryotes.

As an alternative to these co-evolution studies based on individual genes, a number of recent studies have independently proposed a different approach based on the correlation of homologous gene group sizes across genomes. Ranea and colleagues [13] grouped protein encoding genes hierarchically in families based on their 3D structure (CATH structural domains) and sequence similarities. They then measured the evolutionary convergence between them through *copy number*, referred to here as *group size*, correlation analyses based on a euclidean distance statistic to quantify profile pair relatedness. Cordero and coworkers [14] studied the co-evolution between pathway co-occurring gene families from the COG database [15]. They introduced a more sophisticated phylogeny-aware approach based on the reconstruction of the histories of gene content across phylogenetic lineages. Thus, gene families (or groups) that showed concerted changes in size at the same branches along the species tree are predicted to have co-evolved. Despite their methodological differences, both studies reached the conclusion that considering gene group size instead of total presence or absence of a particular gene can lead to a considerable improvement in predictive performance, at least in their respective datasets. In addition, very recently Tuller and colleagues [16] applied non-parametric correlation analysis of gene group size changes along branches of the species tree to elucidate functional relationships between GO terms. They used this approach in conjunction with correlation of evolutionary rates to reconstruct a global gene co-evolution network in yeast species.

Although all these approaches have been used successfully in a certain number of studies, no objective comparison has been performed to evaluate their relative performance, particularly in the more complex situation of eukaryotic genome studies. Here, we have constructed a benchmark containing both positive examples of functionally related gene pairs and negative examples of unrelated pairs, based on the functional relationships defined in the STRING database [17]. The benchmark contains examples of two different types of relationships: physical protein-protein interactions (PPI) and co-occurrence in the same cellular pathway (CoPW). We have used this benchmark to perform a comprehensive study of "classical" gene presence-absence phylogenetic profiles, as well as gene group size based analyses taking into account the species phylogeny, in order to measure the ability of each technique to predict these functional relationships in the eukaryotic life domain.

## Methods

### Gene group test sets in eukaryotes based on orthology and shared domains

**Figure 2:**
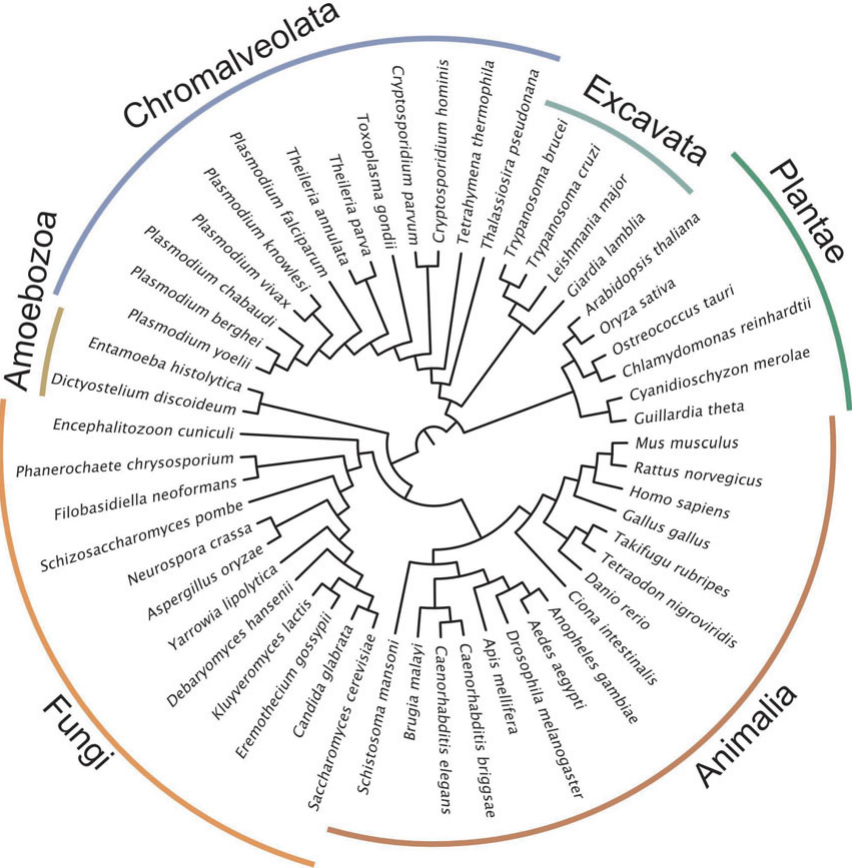
**Species tree of the 53 eukaryotic species used in the study**. Although the real position of the last common ancestor (LCA) of all eukaryotes remains unclear, we chose a plausible location at the root of Unikonta (here represented by animals, fungi and amoebae for analyses that required a rooted tree. The figure was built using FigTree v1.2.2 http://tree.bio.ed.ac.uk/.

The first step in any phylogenetic profiling analysis is the definition of homologous gene groups in the set of species under study. Here, we have used a large test set of eukaryotic orthologous gene groups extracted from the OrthoMCL database version 2 [18]. This orthology prediction database offers a broad eukaryote species sampling and has performed well in gene function based benchmarks [19]. Each orthologous group may contain an arbitrary number of paralogs within each species as determined by the OrthoMCL multi-species algorithm [20], typically resulting from post-speciation gene duplications. Therefore OrthoMCL groups are appropriate for the evaluation of both presence-absence and gene group size based approaches as functional link predictors in eukaryotes at the domain level. The resulting test set includes a total of 53 eukaryotic species (Figure 2) and 54228 orthologous gene groups present in at least two of these species.

Since the OrthoMCL group granularity may be too fine for group size based analyses to be effective, we also generated a coarser gene grouping based on InterPro database [21] domain and family level matches across all eukaryotic proteins in OrthoMCL (using the precomputed matches available on the OrthoMCL database website). This second approach frequently leads to genes being classified in more than one group due to the presence of multi-domain or fusion proteins. Despite the fact that such co-occurrence may in fact be a good indication of functional relationship between groups (the *rosetta-stone *method [22]), it obscures the evaluation of the real merit attributable to the phylogenetic profiling approach itself. Therefore, we excluded from our analyses, profile comparisons between gene groups where the size of their intersection was greater than 1% of the size of the smaller group. Any effects due to group overlap were considered negligible below this threshold. The resulting set includes 4378 groups present in at least two eukaryotic species. Table 1 shows some general statistics relevant to this study concerning the groups generated using OrthoMCL and InterPro based approaches.

**Table 1:** General properties of homologous gene groups.

	OrthoMCL	InterPro
Conserved groups	8999	2716
Group size^(1)^	27.01 ± 38.06	143.74 ± 392.84
Size per species^(1)^	0.51 ± 0.72	2.71 ± 7.41
FN_UW_/(FN_UW_+TN_UW_)	0.0061	0.084
FN_W_/(FN_W_+TN_W_)	0.026	0.49
Clustering Coefficient^(2)^	0.295	0.516
Characteristic path length^(2)^	2.790	2.003

^(1)^Cells show mean and standard deviation.

^(2)^Calculated on the functional link network between groups present in STRING database.

FN = False Negative, TN = True Negative

### Eukaryotic phylogeny and selection of conserved gene groups

In order to apply more accurate genome phylogeny based profiling methods, we needed to specify a fully resolved species phylogeny for the 53 species under analysis. We used a curated phylogeny (Figure. 2) taking into account the current understanding of deep evolutionary relationships between eukaryotic taxon groups [23-27]. The root of the tree was placed at a plausible location corresponding to the last common ancestor of all eukaryotes: the split between unikonts (amoebozoa, fungi and metazoa) and bikonts (platae and the rest of protists) [28]. In order to focus on functional relationships that may be relevant to most of the eukaryotic species examined, we considered only those gene groups that expand across these two major taxon groups. The final dataset consisted of 8999 OrthoMCL and 2716 InterPro groups.

### Gene presence-absence profiling

**Figure 3:**
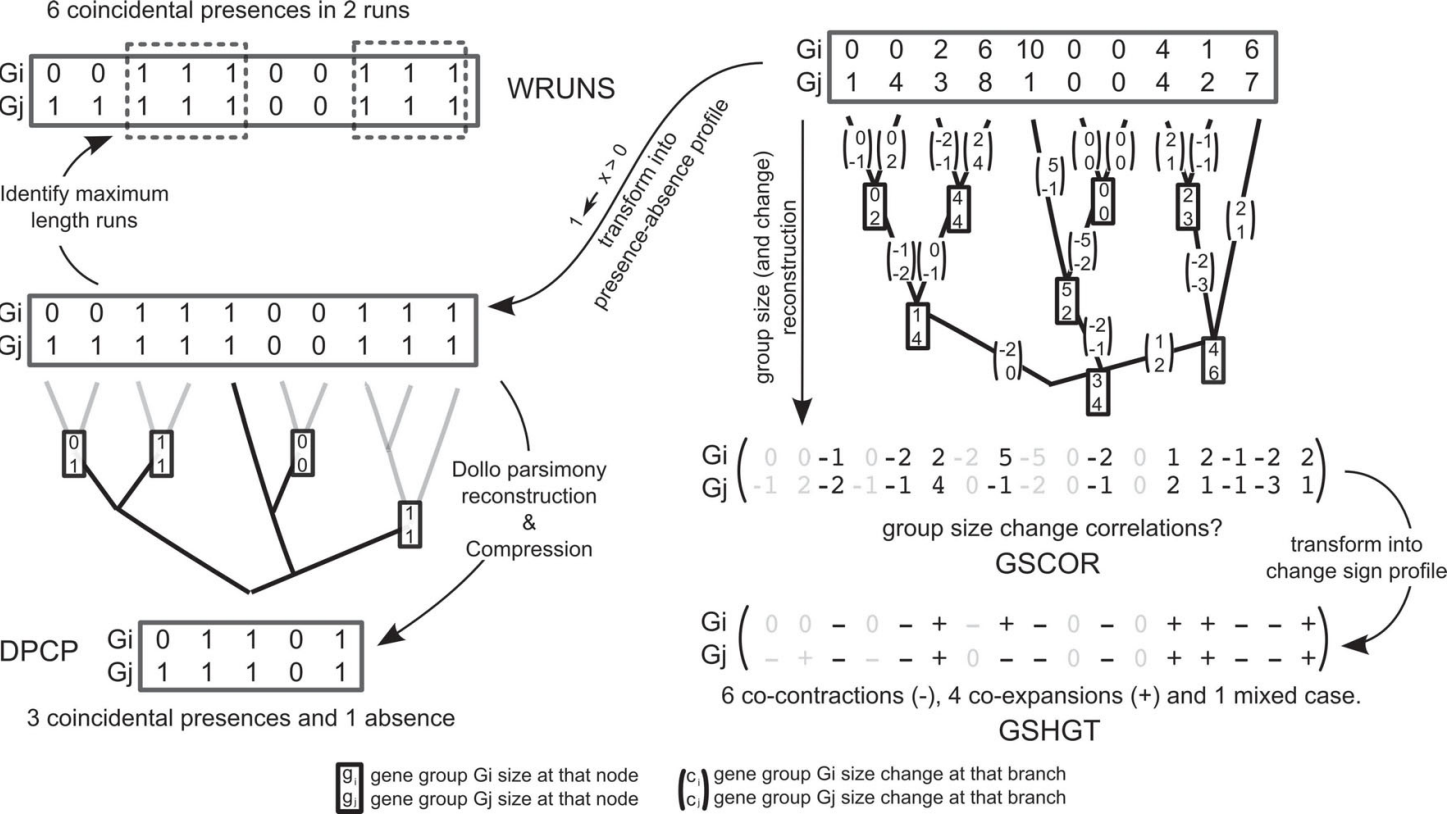
**Graphical summary of species tree aware approaches evaluated in this manuscript**. On the left hand side, classical presence-absence profiling. First, for each profile pair (*G*_*i *_and *G*_*j*_), gene group counts are converted to a binary representation. The significance of co-occurrences is then evaluated using 2 different methodologies (DPCP and WRUNS). The gray branches in the DPCP tree indicate compressed subtrees (see main text for details). On the right hand side, gene group size based methods. First, ancestral group sizes are reconstructed, and the group size change at each branch is calculated (see main text for details). Then, profile pairs are tested for evidence of co-evolution (GSCOR and GSHGT).

For each of the groups in the 2 datasets described above, a presence-absence profile was constructed, with each position corresponding to the 53 species under study. The position in the profile was set to 1 if the species was present in the orthologous group, regardless of the number of genes, and was set to 0 if the species was absent. The similarity of each pair of profiles was then measured using two different approaches: (i) co-occurrence of group loss as inferred using Dollo parsimony and (ii) weighted-hypergeometric and runs-corrected scores. These two approaches are described in more detail below and in Figure 3.

### Presence-absence profiling based on Dollo parsimony inferred gene losses

Dollo parsimony was initially conceived as a tool to infer phylogenies [29], but recently some studies have applied this approach to the analysis of the evolution of gene content in genomes and functional linkage between proteins in eukaryotes [30-32]. Dollo-parsimony assumes that of two possible state transitions, in our case, presence to absence and *vice versa*, one is more probable than the other. In the current context, we assume that gene gains can only occur once, but that genes can be lost independently in many lineages. Thus we assumed that recurrent gain of the genes in different eukaryotic lineages via lateral gene transfer (LGT) is relatively small in comparison to the number of genes inherited vertically.

In order to measure the statistical significance of the similarities observed between two profiles, we applied the hypergeometric test based on the four possible result classes (i.e. combinations of presence and absence) at each position on the profile. We also implemented the phylogenetic correction used in the STRING database by compressing subtrees with all extant taxa in the same result class into a single observation (described in [8]). Additionally we bound trees at branches where a gene group loss occurs in either of the two orthologous groups. We considered that two gene groups cannot influence each other's evolution long after one of them has been lost (i.e. after the next speciation event in the species tree under study). In the rest of the article we refer to this method as DPCP (Dollo-parsimony compressed profiles).

### Presence-absence profiling using ordered profiles and runs-based scoring

This is the approach proposed by Cokus and coworkers [7]. In summary, their approach scores the number of coincidental co-presences (1 and 1) in a pair of group profiles, taking into account the proportion of gene groups present in each genome (weights) and the species phylogeny. The scoring procedure does not consider the tree topology *per se*, but it does so indirectly by ordering the species in the profiles according to their evolutionary relationships and then counting the number of *runs *(i.e. maximal consecutive co-presence positions) between two profiles (Figure 3). Although not equivalent, the number of runs is positively correlated with the combined number of gene group gains and losses in both profiles and thus the *information content *present in the profile pair. For example, multiple co-presences found within a small number of runs may indicate gene conservation in lineages rather than co-evolution, whereas the same number of co-occurrences in a larger number of runs is a more convincing indicator of true co-evolution.

The number of observed runs and the resulting score depends on how we graphically sort descendant subtrees at each ancestral node in the species tree. We chose a single order for all comparisons following the approach indicated in the original paper [7], which involves minimizing the genome content dissimilarities between adjacent genomes in the profile. From this point on we refer to this method as WRUNS.

### Gene group size profiling

In gene group size based profile approaches, instead of coding gene groups according to presence and absence, the exact number of gene group members at each genome is preserved at each position. Therefore there is no loss of information at this point. Methods that do not take into account the phylogeny (e.g. [13]) could compare the profiles with no further changes. However, phylogeny aware methods include an additional step where profiles "unfold", by incorporating an additional position for each ancestral taxon or internal node in the species tree that contains an estimated ancestral group size. Then, the group sizes in the profile are transformed into the final signed size change along the branch that lead to them (Figure 3 right). This is calculated as the group size at that node minus the group size at the direct ancestor on the tree. As a result, each position in the final profile, representing a branch in the species phylogeny rather than an extant species, includes a *neutral *sign or 0 if there was no size change, a negative number if the gene group decreased in size, *contraction*, or a positive number if its size increased, *expansion*.

### Reconstruction of ancestral gene group sizes

A key element in these approaches is to infer ancestral gene group sizes. Here we used two different approaches: a parsimony based reconstruction method and a new approach based on gene trees.

For the parsimony based reconstruction, we implemented the algorithm described in Cordero et al 2008, who minimized a cost function on the minimum number of genetic events (individual gene losses, duplications or gains) necessary to explain the observed pattern of group sizes in extant species. Here, we used the cost function *F *(Eq. 1); in this function we do not consider *de novo *gene gains, that would result from LGT, thus increases in gene group size can only be due to existing gene duplications. We adjusted the gene duplication, *d*, to loss, *l*, cost ratio parameter, μ, using observed relative frequencies using the gene tree based reconstruction approach (μ = 2.5).(1)

The novel gene tree based approach involves the reconstruction of the genealogy for each gene group and the mapping of gene gains and losses onto the species tree. To do this, we used the MAFFT software [33] to construct multiple sequence alignments for each gene group. Then we used the TreeBest package [34], first to build the corresponding trees using the neighbour joining (NJ) algorithm on multiple substitution corrected amino acid distances, and second to map genetic events onto branches on the species tree.

Often protein domain rearrangements and fast sequence evolution hampers sequence alignment and in turn gene tree reconstruction. Consequently, we needed to split each gene group into subgroups more amenable to alignment and tree inference. In order to do this, we performed a greedy decomposition in maximum *cliques *using Cliquer [35]. Here we define a clique as a subset of genes whose sequences are aligned with any other member of the clique over at least 50% of the length of the shortest sequence in the pair. This threshold was determined in tests on a subset of groups and permits reconstruction of subgenealogies without over-fragmentation of the subfamilies. At each decomposition step, the maximum clique is subtracted from the remaining set of genes until only one or zero remains. Since calculating maximum cliques is a computationally demanding problem (NP-hard in complexity theory terms), we used Nmclique [36], an approximate heuristic solver, as long as the remaining group size was greater than 300 genes. Finally, once a gene group was fully decomposed, TreeBest was run on each subgroup and inferred genetic events were merged by adding them up on the corresponding branches of the species tree.

### Group size based profiling significance testing

We applied two methods to identify gene group size change profile pairs that show a significant level of coevolution: first using a correlation test on unmodified size change values and second using a hypergeometric test on the distribution of the sign of the change (expansions and contractions) across branches. We refer to these as GSCOR (correlation test of group size change) and GSHGT (hypergeometric test of group size change) in the text from now on. Subscripts (_Pars_) and (_GeneT_) indicate that ancestral group size were reconstructed using parsimony or gene trees respectively (Table 2).

**Table 2:** Summary of methods and abbreviations.

**Abbrev**.	Methodology	Statistic
DPCP	Presence-absence profiling using Dollo parsimony compressed profiles.	Hypergeometric p-value
WRUNS	Presence-absence profiling using Weighted hypergeometric and runs test	Weighted hypergeometric score
GSHGT_Pars_	Parsimony reconstructed ancestral group sizes	Hypergeometric p-value
GSCOR_Pars_	Parsimony reconstructed ancestral group sizes	Kendall-τ p-value
GSHGT_GeneT_	Gene-tree reconstructed ancestral group sizes	Hypergeometric p-value
GSCOR_GeneT_	Gene-tree reconstructed ancestral group sizes	Kendall-τ p-value

For GSCOR we implemented an approach similar to the one proposed by Cordero and colleagues [14] but opted for a non-parametric method, Kendall-t ranks correlation coefficient and p-values as changes are not necessarily normally distributed. We also tested a partial correlation correction to take into account the bias effects of heterogenous genome sizes. However this did not result in an improvement in predictive performance (data not shown) thus we opted for the uncorrected version, relying on empirical thresholds and the p-value fitted distribution to eliminate this and other biases as described in Results and Discussion.

In both approaches we did not take into consideration *ambiguous *branches that exhibit no group size change. This allows a binary encoding of expansions and contractions in GSHGT and avoids numerous ties of 0 in GSCOR.

### Measure of profile pair information content

For the GSCOR method, we used the number of branches considered in the test, i.e. those with expansions and contractions in both profiles, to quantify information content. In contrast, for DPCP, WRUNS and GSHGT, we used the geometric average of the profile entropies multiplied by the length, *l*_*ij*_, of the profile pair under consideration:(2)

The individual profile entropy *H *is calculated using the fraction of positions for each possible symbol (DPCP {0,1} and GSHGT {-,+}):(3)

### Calculation of information content adjusted profile pair scores

We used two approaches that take into account IC, in order to classify profile pairs as putatively co-evolving or not. First, we used a single cut-off threshold based on a negative control set built as described below. Second, we fitted a bivariate distribution of scores versus information content for each method using the kernel approach implemented in the MASS R package (function kde2d).

### Positive and negative test sets

We used the STRING database version 7 [37] as a unified source of both experimentally verified and computationally inferred protein-protein functional relationships. We built two different categories of true positive (TP) datasets. The first set, TP^CoPW^, contained gene pairs that participated in the same KEGG cellular pathway [38] as stored in STRING. The second set, TP^PPI^, included interacting protein pairs from interactome databases present in STRING (see [37] for details). We propagated the relationships defined for individual genes to gene groups, if there were at least two disjoint pairs of genes in each group that were functionally related to each other. In the case of TP^CoPW^, at least one pair must belong to a unikont species and the other to a bikont. This way we intrinsically give more weight to functional links that are more likely to exist in most eukaryotes. Due to limited species sampling of protein-protein interactions, in the TP^PPI ^we included all group relationships that had evidence in both vertebrates and non-vertebrates.

In order to construct the negative control dataset, we wanted to explicitly exclude potential functionally linked gene pairs and their corresponding gene groups. We therefore built a "false negative" (FN) set of related gene pairs based on the absence of any kind of evidence in STRING, except functional links only supported by the phylogenetic profiling method implemented therein; if included, these would result in an overestimation of the performance of the phylogenetic profiling methods under evaluation, due to similarities between the criteria used to build the negative control set and to compare their profiles later. Finally, we propagated all gene level relationships to their respective OrthoMCL and InterPro groups with no additional restrictions.

In each analysis we generated a putative true negative set TN (200,000 pairs). To do so, we constructed random gene group pairs and discarded those present in the FN set until we reached the targeted size. Note that all TP pairs are included in the FN set, therefore TP pairs are also automatically excluded from the negative control.

We used two approaches for generating random pair samples: based on a weighted and unweighted group random pair generator. In the unweighted approach, resulting in the pair set TN_UW_, each group has the same probability of being picked, whereas the weighted approach gives a weight to each group proportional to the number of gene members, resulting in the pair set TN_W_. The motivation behind using a parallel weighted approach is the noticeable bias in the orthologous and domain level group sizes found in the TP sets in comparison with unweighted TN_UW _sets. In order to generate positive predictive value (PPV) plots, we estimated the real proportion of TP group pairs using the number of unweighted random pairs rejected because they were found in the FN set.

## Results and Discussion

A total of six phylogenetic profiling approaches described in Methods were tested based on the positive and negative datasets of functionally linked genes or gene groups. These included two methods based on the traditional presence-absence profiles of individual genes: a full species phylogeny based method (DPCP) and a computationally efficient heuristic method (WRUNS). In addition, four different gene group size approaches were tested resulting from the combination of two methods to infer ancestral gene group sizes (parsimony and gene tree) and two methods to evaluate the significance of the co-evolutionary signal (GSCOR and GSHGT). A summary of the methods and abbreviations used is shown in Table 2.

### Sensitivity of presence-absence profiling at the orthologous group level

We first applied the traditional presence-absence profiling methods, here represented by DPCP and WRUNS, to the test set of OrthoMCL orthologous groups. The goal was then to correctly predict known relationships extracted from the STRING database, namely co-presence in pathways (TP^CoPW^) or protein-protein interactions (TP^PPI^). To measure the overall sensitivity of the methods, we initially defined a single global p-value or score threshold for each method using the distribution of values observed in the negative control sets at the typical standard type-I error level of 5% (i.e. 95% specificity).

The first two columns in Table 3 show the resulting sensitivities. DPCP is more sensitive with a maximum of 20.7% true positives versus 14.9% for WRUNS on the TP^CoPW ^test set, using TN_UW _as a negative control. Similar results are obtained for TP^PPI ^with sensitivities of 17.5% and 13.7% respectively. Using the weighted negative control, TN_W_, the two approaches achieve more similar but slightly lower sensitivities (Table 3). Thus, in this case, we conclude that the negative test set sampling approach has little effect on the sensitivity estimates.

**Table 3:** Sensitivity of co-evolution detection methods on OrthoMCL profile pairs.

Positive pair set	Presence/absence profiling		Group size profiling			
	DPCP	WRUNS	GSHGT_Pars_	GSCOR_Pars_	GSHGT_GeneT_	GSCOR_GeneT_
Unweighted negative control (TN_UW_)						
TP^CoPW^	20.7%	14.9%	6.4%	12.2%	17.3%	18.6%
TP^PPI^	17.5%	13.7%	17.2%	25.9%	33.7%	37.9%

Weighted negative control (TN_W_)						
TP^CoPW^	16.3%	14.3%	6.4%	12.5%	13.8%	10.5%
TP^PPI^	15.1%	13.3%	17.2%	25.9%	29.0%	27.2%

**Figure 4:**
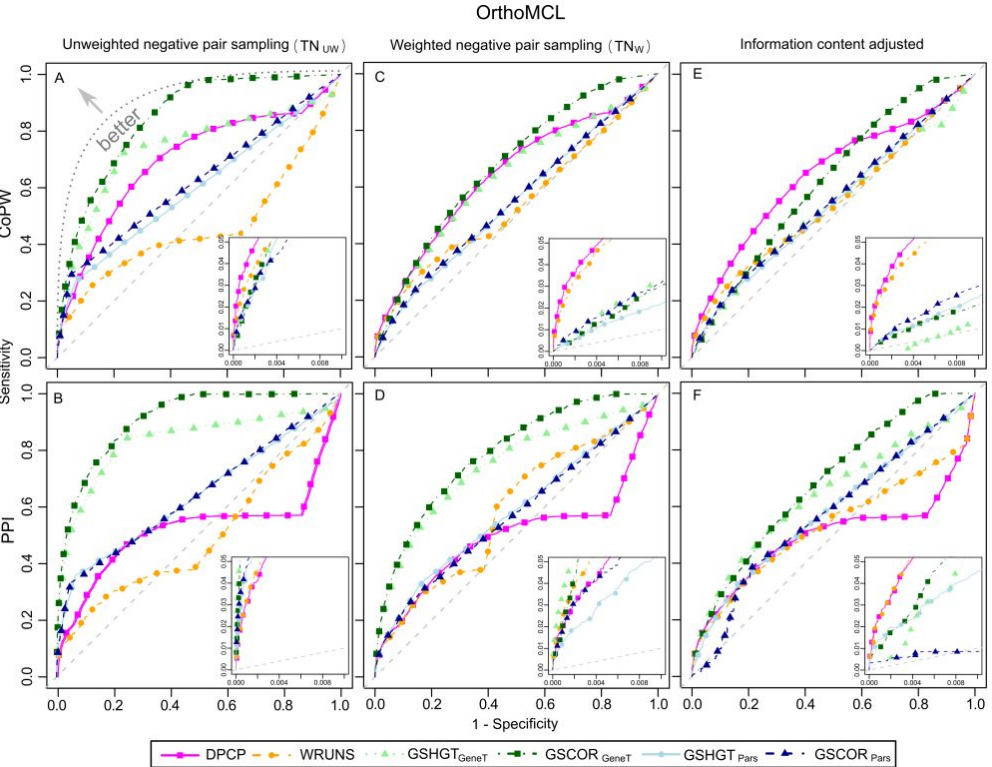
**ROC curves of different methods applied to OrthoMCL based gene groups**. The sensitivity (x-axis) is evaluated using TP^CoPW ^(top: A, C and E) and TP^PPI ^(bottom: B, D and F) whereas specificity (x-axis) is evaluated using TN_UW _(left: A and B), TN_W _(centre: C and D) and information content adjusted p-values (right: E and F). Inset graphs give a zoomed in view of the high specificity - low sensitivity regions (top scoring cases).

The corresponding ROC curves (Figure 4 pink and yellow), which plot sensitivity versus specificity, show that these methods are better than random classifiers especially in the region of high scoring cases (inset diagrams). Nevertheless, both presence-absence profiling approaches sometimes fall just above or below the diagonal, generally showing a poorer performance on the PPI positive set compared to the CoPW set. This is due to a considerable percentage of profile pairs that do not exhibit enough variability or number of genetic events (information content), resulting in an elevated number of degenerated maximum and non-significant p-values (= 1) that affect CoPW and PPI (especially the latter).

### Sensitivity of group size based profiling approaches at the orthologous group level

Since the OrthoMCL orthologous groups may contain a variable number of paralogs for each species, group size based approaches are also applicable in this scenario. Accordingly, we also evaluated their sensitivity using the same procedure as for the presence-absence profiling methods (Table 3). In order to calculate empirical thresholds, we only considered group pairs with a minimum number of 10 common branches that have undergone a group size expansion or contraction, since lack of either group size variability across species or phylogenetic spread renders a considerable number of profile pairs unsuitable for this purpose.

The ancestral gene group size reconstruction using Parsimony also yielded results better than a random classifier based on the ROC curves (Figure 4, light blue and dark blue). They were roughly equal to or inferior to presence-absence methods in the high selectivity region close to the origin of the axes with the exception of the TP^PPI ^using unweighted negative test set sampling (zoomed in insets in Figure 4). Using GSCOR_Pars_, the observed sensitivities are higher than with GSHGT_Pars _(Table 3).

In contrast, the gene tree based reconstructions achieved in general a better sensitivity, closer to that achieved by DPCP on the TP^CoPW ^test set and with a noticeable improvement on the TP^PPI ^set using both GSHGT and GSCOR. GSCOR_GeneT _achieves an more than 2 fold increase using the unweighted negative set sampling (37.9% versus 17.5%). Also the corresponding ROC curves (Figure 4, light green and dark green) are the ones closest to the ideal classifier, indicating that these methods seem to perform better overall (Figure 4A-D).

### Sensitivity at the domain sharing and gene family level

In a second experiment, we applied the same analyses to coarser groups of genes from the InterPro dataset (see Methods). These groups are clearly more extensive in comparison with OrthoMCL groups, with a 5 fold larger mean group size (Table 1). A larger number of members allows a greater group size variability across extant and ancestral gene groups, and thus a larger number of informative tree branches that have undergone group size expansions or contractions.

It is worth noting that the weighted negative pair sampling generates a staggering 49% pairs present in the FN profile pair set (see Methods). In other words, if two genes are chosen at random, there is nearly a 50% chance that they contain some functionally related protein domains (i.e. at least one functionally linked protein pair contains these domains). This is not surprising if one considers that even assuming an unrealistic random intergenic functional network, larger gene groups would have an increasing chance to exhibit some direct or indirect functional link through some of its members.

Although presence-absence profiling is not appropriate for coarse gene clusters (since most positions are 1), we applied DPCP and WRUNS to these two datasets in order to compare their performance with gene group size based approaches. Presence-absence profiling clearly loses overall sensitivity when applied to coarse gene groups (Table 4). Moreover, their sensitivity does not even reach the designed type-I error for the prediction of PPI relationships using the InterPro groups. This confirms that presence-absence profiling is less useful at this group granularity level, although it still does better than a random classifier on the CoPW positive profile pair set.

**Table 4:** Sensitivity of co-evolution detection methods on InterPro profile pairs

Positive pair set	Presence/absence profiling		Group size profiling			
	
	DPCP	WRUNS	GSHGT_Pars_	GSCOR_Pars_	GSHGT_GeneT_	GSCOR_GeneT_
Unweighted negative control (TN_UW_)						
TP^CoPW^	11.0%	9.8%	13.1%	18.7%	18.8%	31.9%
TP^PPI^	3.1%	3.3%	27.5%	34.4%	39.7%	63.7%

Weighted negative control (TN_W_)						
TP^CoPW^	12.0%	11.9%	9.2%	13.9%	11.7%	14.4%
TP^PPI^	3.5%	3.9%	21.6%	26.5%	29.2%	42.4%

In contrast, using the group size based approaches, the sensitivity remains equal or increased with coarser clusters of genes. The only exception is the gene tree reconstruction with the hypergeometric test (GSHGT_GeneT_) for the prediction of CoPW relationships, where sensitivity slightly decreased (approx. 2% less). The maximum sensitivity achieved is as high as 63.7% for the prediction of PPI using the InterPro groups and the unweighted negative control sampling to calculate thresholds.

**Figure 5:**
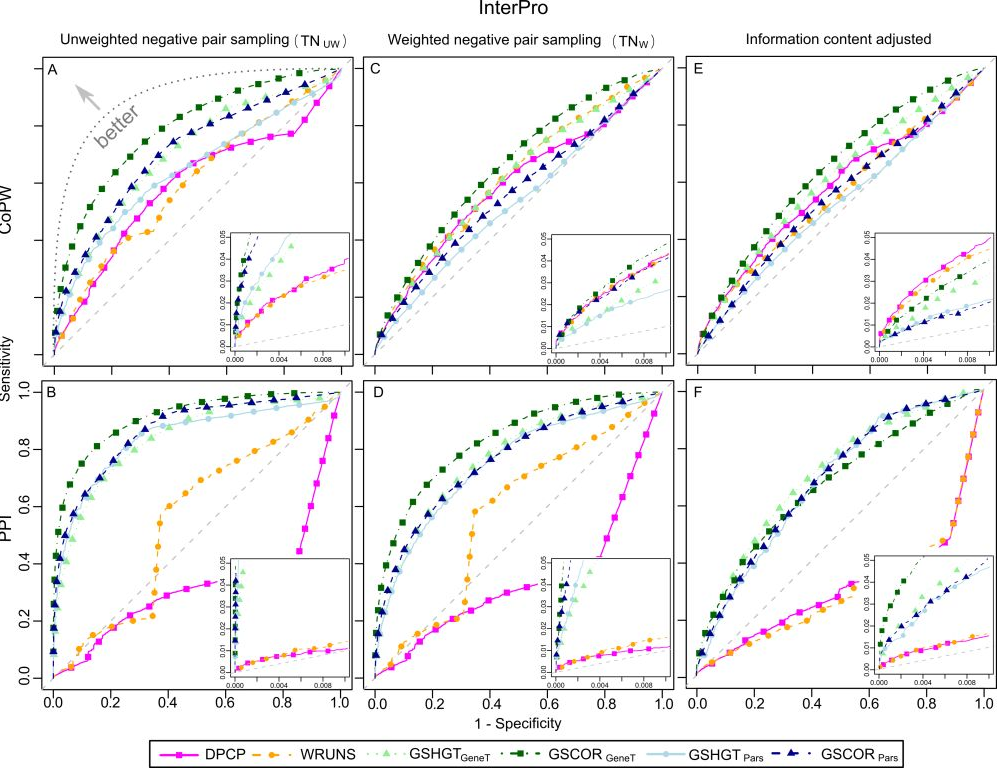
**ROC curves of different methods applied to InterPro based gene groups**. The sensitivity (x-axis) is evaluated using TP^CoPW ^(top: A, C and E) and TP^PPI ^(bottom: B, D and F) whereas specificity (x-axis) is evaluated using TN_UW _(left: A and B), TN_W _(centre: C and D) and information content adjusted p-values (right: E and F). Inset graphs give a zoomed in view of the high specificity - low sensitivity regions (top scoring cases).

The corresponding ROC curves are consistent with these results (Figure 5A-D). These show an appreciable decrease in performance of the presence-absence profiling methods compared to the results obtained with the OrthoMCL groups (see Figure 4), especially for the prediction of PPI positive pairs. The group size based approaches using parsimony reconstruction (light blue and dark blue curves) improve considerably in most cases (except for CoPW predictions using weighted negative sampling), in contrast to the gene tree based reconstruction (light green and dark green curves) that only results in a noticeable improvement for the PPI predictions.

### Effect of information content on sensitivity and false positive rates

**Figure 6:**
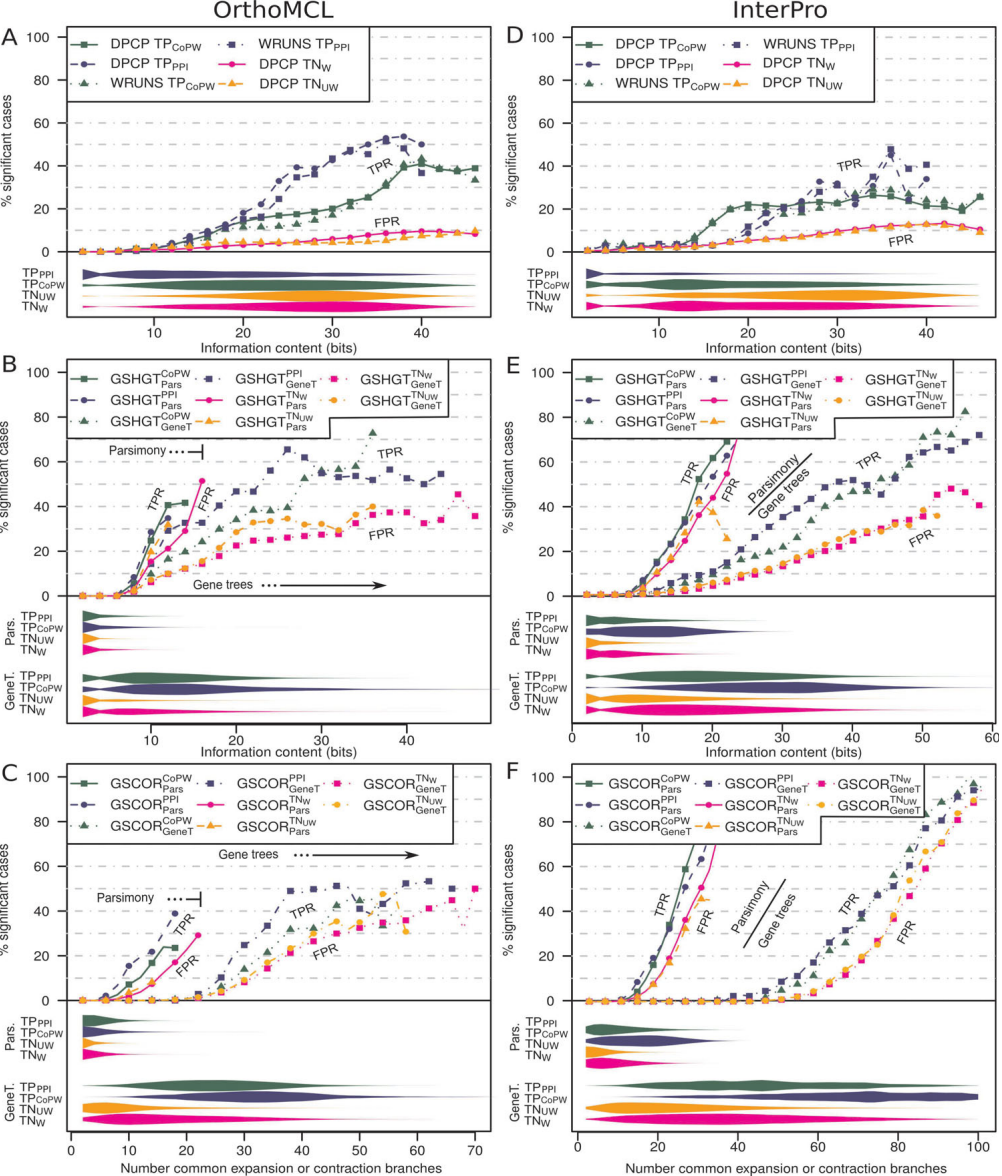
**Sensitivity (TPR) and false positive rate (FPR) plots versus profile pair information content (IC)**. Here we use a single type-I error threshold value of 5% and TN_W _as a reference. Left column (A-C) corresponds to OrthoMCL groups and the right column (D-F) corresponds to InterPro groups. The top row (A and D) shows results using presence-absence profiling approaches, the middle row (B and E) represents group size approaches using the hypergeometric test (GSHGT), and the bottom row (C and F) corresponds group size approaches using the Kendall-t correlation index (GSCOR). Results for WRUNS on TN_W _and TN_UW _are omitted since they are extremely similar to DPCP's. Violin plots under each diagram indicate the frequency distribution of profile pairs across information content (wider plots indicate higher frequencies). Each colour represents a different positive, TP^PPI ^(blue) and TP^CoPW ^(green), or negative, TN_UW _(magenta) and TN_W _(orange), profile pair dataset. Thus the first two represent sensitivity or true positive rate (TPR) whereas the second two indicate FPR using alternative negative control sampling approaches. Sensitivity, FPR and IC frequency distributions are smoothed by considering neighbouring data points along the y-axis (radius 2) with equal weights. Data points with less than 10 observations are not shown.

To evaluate to what extent information content (IC)may have an impact on the performance of the different methods, we plotted sensitivity, or true positive rate (TPR), and false positive rates (FPR) at a standard type-I error level of 5% against a measure of profile pair IC (described in Methods) (Figure 6). All approaches show better performance for the prediction of PPI compared to CoPW relationships. Moreover, the sensitivity is in most cases higher than the FPR, although the ratio between the two quantities is lower using the group size methods.

For the gene presence-absence methods applied to OrthoMCL groups (Figure 6A), the sensitivity increases steadily with increasing IC clearly above the FPR. Both DPCP and WRUNS achieved similar performances at the same IC levels. The sensitivity is clearly higher for the prediction of PPI compared to the CoPW dataset. At the 5% type I error level, sensitivity exceeds 50% in the highest informativeness regions. In contrast, the FPR barely reaches 10% under the same conditions, thus resulting in an approximate 5 fold ratio between sensitivity and FPR.

For the group size based approaches (Figure 6B and 6C), the parsimony ancestral size reconstruction apparently results in lower IC (measured by the number of branches that exhibit expansion or contraction in both profiles simultaneously). Nevertheless this observation is not by itself a clear indicator of the overall performance superiority of the gene tree based reconstruction. At best, for the parsimony reconstruction with the hypergeometric test (GSHGT_pars_), FPR reaches values as high as 30% with a contrasting sensitivity that only occasionally surpassed the 40% mark (Figure 6B). Using gene tree based reconstruction, these figures reached 35-40% and 65-70% respectively. In general, sensitivity vs FPR in group size based approaches does not go far beyond the 2 fold mark. In contrast, the correlation based approach (GSCOR) using either parsimony or gene tree based ancestral group size reconstruction does not show such a clear distinction graphically between CoPW positive and negative control sets specially using gene tree reconstruction (Figure 6C). The performance is slightly better for PPI positives.

The sensitivity and FPR of the methods applied to InterPro groups (Figure 6D-F) are very similar to the plots obtained using OrthoMCL groups from a qualitative point of view. Nevertheless there is a clear loss of sensitivity in presence-absence methods at this group level (Figure 6A and 6D). The differences between the group size based approach curves (Figure 6B vs E and C vs F) are mainly due to differences in the IC ranges and thresholds used.

### Effect of information content heterogeneity

In order to investigate the IC ranges for the profile pairs in each of the test sets, we plotted the relative profile pair IC frequency distribution along the x-axis (violin plots under each diagram in Figure 6). In all approaches and gene group granularities, the regions with high frequency in the positive profile pair test set do not match the maximum sensitivity (always associated with high IC). This seems to be the main factor that limits profiling performance. Additionally, the profile pair distribution across IC levels is clearly different between the positive and negative datasets in each possible combination of profiling methodology and gene group granularity. Using presence-absence profiling, the positive set frequencies are shifted towards low IC in contrast to the negative control set frequencies, whereas this trend is reversed using group size approaches. This may explain the contrasting performance of presence-absence profiling and group size based profiling within and across different gene group clustering granularities.

In the case of presence-absence profiling, most PPI links are found in the region with less informative profile pairs especially in the InterPro gene group positive test set (Figure 6D). Thus, despite the fact that sensitivity is generally higher when compared to CoPW positive pairs and that PPI functional links should exhibit a stronger interdependence, this type of profiling performs worse in practice (Tables 3 and 4).

These results suggest that we could aim to improve functional link prediction accuracy by using alternative grouping criteria that maximize IC. For example, previous studies in prokaryotes and eukaryotes indicate a gain in performance when considering several similarity thresholds [13,39] as opposed to a single gene clustering criterion. However altering granularity in order to increase the chance of detection by optimising IC may render the results less amenable to interpretation, as cluster boundaries may not correlate with clear biologically interpretable gene groups (e.g. orthologous groups, gene families, shared domains, gene ontology clusters and so forth).

Alternatively, profile pairs could be sorted based on IC for further investigation using potentially more accurate but computationally demanding approaches, such as maximum-likelihood based methods [5]. This would avoid running computationally demanding analyses on profile pairs with low IC that *a priori *are much less likely to show any evidence of coevolution.

### Score adjustment based on information content

In view of the differences in information content (IC) distributions for the positive and negative test sets, even when using weighted negative sampling, we reassessed the performance of the different approaches after correcting for IC heterogeneity on a case by case basis. Taking TN_W _as a control and using a kernel fit, we estimated the conditional probability distribution of the scores given the IC under the null hypothesis that gene groups are not functionally related: Pr*(score|IC*_*ij*_*)*. Then, the scores were converted to adjusted p-values equal to the probability of obtaining a score equal to or greater than the observed score given the IC found in the pair. The resulting profile pair p-values should more accurately reflect our confidence in the prediction of co-evolution of the corresponding gene groups. The plots on the right-hand size in Figures 4 and 5 show the adjusted ROC curves next to their non-adjusted counterparts using unweighed and weighted negative sampling (left and middle plots respectively) and Table 5 shows the sensitivity estimates at the type-I error level of 5% (p-value <= 0.05) for the IC-adjusted p-values.

**Table 5:** Sensitivity of Information Content adjusted p-values at 5% type I error using kernel fit.

Positive pair set	DPCP	WRUNS	GSHGT_Pars_	GSCOR_Pars_	GSHGT_GeneT_	GSCOR_GeneT_
OrthoMCL						
TP^CoPW^	16.4%	13.6%	0.2%	2.2%	5.7%	6.5%
TP^PPI^	17.0%	14.5%	1.0%	0.9%	14.4%	14.8%

InterPro						
TP^CoPW^	10.9%	10.7%	2.0%	5.3%	8.8%	11.1%
TP^PPI^	3.3%	3.9%	4.4%	10.4%	14.3%	16.8%

Note: cases with information content equal to zero are always treated as insignificant. Thus sensitivity can be much lower than type I error (5%).

Our results indicate a decrease in the performance of the gene group size based approaches using IC corrected p-values, while the performance estimate of the presence-absence approaches remains relatively unchanged compared to the single empirical threshold approach. The parsimony reconstruction based analyses are the most affected by the adjustment, particularly when applied to the InterPro datasets, where sensitivities that were previously estimated to be as high as 34% percent (Table 4) decrease to around 10% or lower (Table 5). All in all, the group size based approaches seem to lose most of the advantage they had with respect to presence-absence profiling when the co-evolutionary signal is measured objectively based on the IC of the profile pair. In other words, we overestimate the performance of gene group based approaches unless, either the profile pair scores are adjusted for IC, or the statistical differences between the positive and the negative control test sets accurately reflect the properties of the real world data.

Thus, the typical approach of generating unweighted random pairs to build a negative dataset and a single cut-off value results in a very clear overestimation of the accuracy of gene group size approaches. Performance improvement using this strategy does not seem to be founded on a real gain of co-evolutionary signal as much as statistical biases due to heterogeneous distribution of informativeness between positive and negative control groups. Jothi and colleagues [10] made an important remark on the importance of a careful selection of the null hypothesis in order to avoid spurious results due to profile heterogeneity. They focused their discussion on the effect introduced by combined analyses of profiles with different phylogenetic spread. The adoption of species tree aware approaches, such as the ones used in this study, together with the restriction of considering only groups that are present on both sides of the root of the tree, should reduce these biases. Nevertheless, differences may still remain and can result in artefactual detection of functional relationships between gene groups.

It should be noted however that the inability to distinguish between putative functionally linked and unrelated pairs may result in part from the difficulties in obtaining a pure negative control dataset totally devoid of functionally linked groups. For instance, weighted negative sampling generates a high proportion of false negatives: 49%. Moreover, the functional network resulting from the clustering of genes into coarse groups is inevitably very condensed, with most genes connected by one or only a few direct functional links (Table 1). Arguably, we should expect to see at least some weak co-evolution between groups that are found close together in the network (i.e. most random pairs).

### Other sources of bias in phylogenetic profiling analyses

For all the methods tested here, the FPR increases for high information content levels. This trend is more pronounced in the group size based approaches and especially when using non-partial correlation corrected coefficients (GSCOR). Previous studies using permutation and simulations have already shown that heterogeneous genome size can affect the outcome of co-evolution tests [13,14]. For example, lineages leading to larger genomes should have a natural tendency to expansions and vice versa. Moreover, genomic scale events, such as whole genome duplications and major genome rearrangements, may introduce additional background co-evolution-like patterns for the affected gene groups that are not a consequence of true functional relationships.

**Figure 7:**
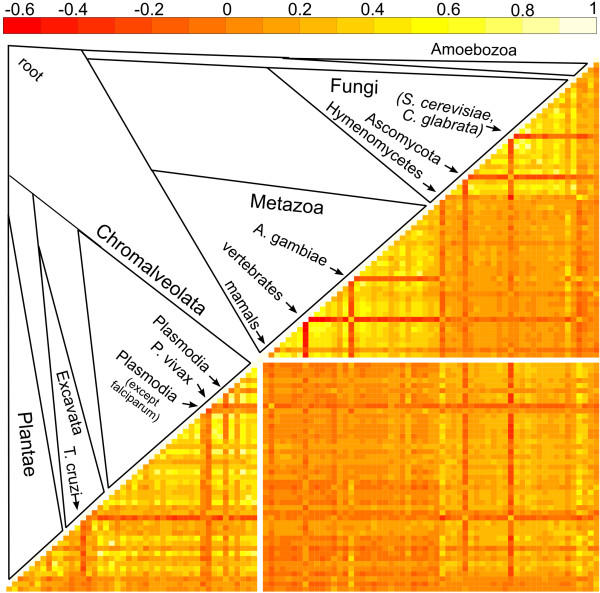
**Heatmap representing partial correlation of reconstructed group size changes between branches on the species tree**. Below the diagonal, each row and column corresponds to a branch in the tree that is schematically depicted above the diagonal. Cell are colour coded according to the partial correlation between branches represented by that row and column (light yellow indicates positive correlation (towards 1), orange tones indicate weak or no correlation (towards 0), whereas red indicates negative correlation (< 0). Partial correlation coefficients were calculated using the TP^PPI ^dataset and the Kendall-t rank index. Above the diagonal, the labelled branches show a contrasting trend in comparison with neighbouring lineages on the species tree.

In addition to genome size effects, we also found evidence for local correlation of group size evolution mostly between neighbouring branches of the species tree (Figure 7). Either positive or negative correlation of group size change between phylogenetic tree branches should result in a number of coincidental expansions and contractions greater than that expected under the null hypothesis, due to the fact that we conveniently (but erroneously) consider observations at different branches or profile positions as independent data points. An evolutionary explanation for this phenomenon might be that closely related phylogenetic lineages tend to follow a similar evolutionary pattern, resulting in positive correlation, and that gene losses are typically preceded by gene gains, resulting in negative correlation between ancestral and successor branches on the tree. Branch evolutionary correlation *heatmaps *such as the one shown in Figure 7 could be used to attain an optimal choice of species for phylogenetic profiling by selecting lineages resulting in a minimization of autocorrelation effects.

### Phylogenetic profiling as a functional link predictor in large-scale analyses

**Figure 8:**
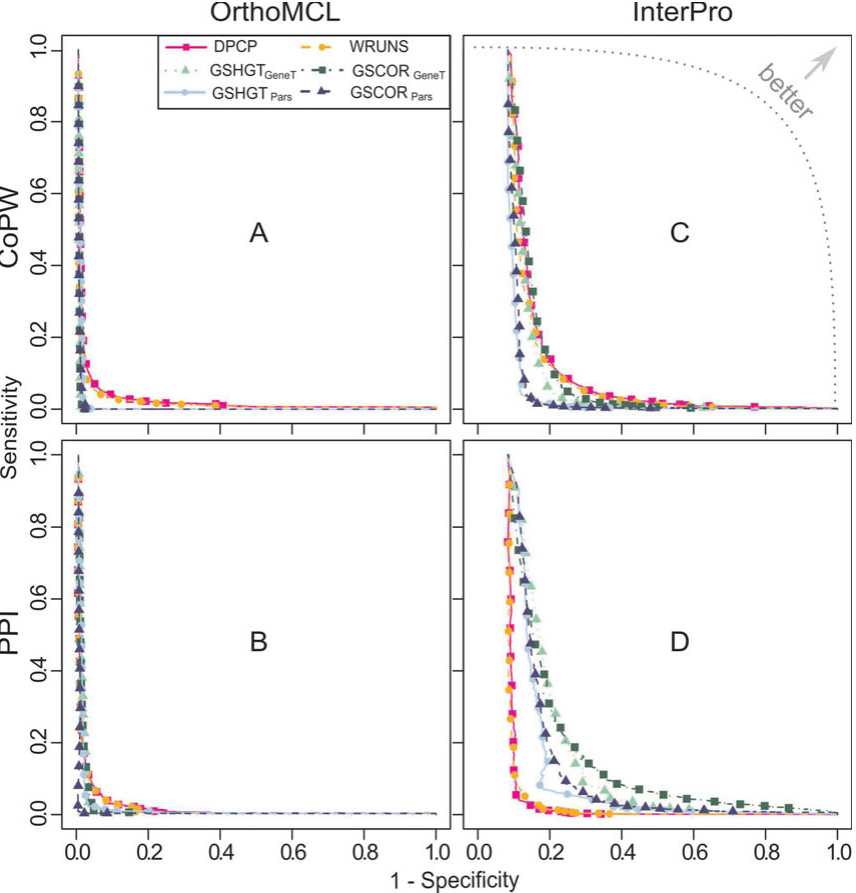
**PPV versus sensitivity plots based on adjusted scores**. Top row shows results using TP^CoPW ^to evaluate sensitivity, whereas the bottom row is based on TP^PPI^. Left and right columns show relative performances for OrthoMCL and InterPro gene groups.

The *positive predictive value *(PPV) is a statistic of special interest for high-throughput functional studies as it quantifies the probability that a positive test outcome is in fact a true prediction. Figure 8 shows PPV plots against sensitivity for analyses using the OrthoMCL and InterPro groups based on IC corrected p-values. Reasonable PPV values (50% or greater) are only obtained at very low sensitivity, especially for the OrthoMCL gene groups (Figure 8A and 8B). This is for the most part due to the low estimated proportion of TP pairs amongst all possible pairs (Table 1) and the fact that only a fraction of these have a high enough IC to be tested.

Consequently, although phylogenetic profiling may be used to unveil some functional relationships with a strong co-evolutionary signal, it is on its own not suitable for large-scale screening of these relationships in eukaryotes. The PPV improves when considering coarser gene clusters (Figure 8C and 8D), but this will lead to subsequent problems in distinguishing true functionally related gene pairs, since the number of possible pairings grows quadratically with the number of members of each group.

### Reassessing the contribution of gene group size based approaches

Gene group size based approaches offer an opportunity to overcome some of the limitations of presence-absence based methods [13,14], for example the analysis of essential gene group profiles (present in all or most species). Nevertheless, even though these methods take in consideration a greater number of genetic events, thus increasing information content, they are still outperformed by presence-absence in certain cases, particularly when applied to the fine-grained OrthoMCL groups (Table 3 and 5 and Figure 4). The most plausible explanation for this is that a total loss of a gene group, without any homologous genes left to perform at least part of its biological role, has a much greater impact than losing or gaining some gene "copies". Thus, common gene group pair co-expansions or co-contractions that do not result in a total gain or loss of the homologous groups under consideration provide less compelling evidence of co-evolution and may introduce noise.

Despite this lack of resolution, the fact that the sensitivity values are typically above the FPR curves (Figure 6) is a clear indicator that these scores are positively correlated with true functional relatedness. Consequently, although group size based approaches may not perform that well in classifying pairs as related or unrelated, they may still be appropriate for defining functional distances between groups and also perhaps for clustering them in functional modules. In any case, high scoring pairs are likely to be true functionally linked groups [13,14]. However, it is preferable to use adjusted scores determined, for example, by the kernel approach described in Methods in order to avoid an over-representation of cases with high IC due to their inherently higher non-adjusted scores.

### Gene trees improve the performance of gene group size change approaches

In an attempt to improve the performance of gene group size based methods, we used gene trees to reconstruct ancestral gene group sizes. This resulted in a clear improvement compared to parsimony reconstruction. Gene trees can map more precisely gene duplications and losses to branches of the species tree using the evolutionary information present in the gene sequences. The total number of genetic events inferred is approximately 9 fold the number recovered using parsimony. Moreover, multiple losses and gains can be simultaneously detected on the same branch, whereas parsimony can only infer an optimal number of gene losses or duplications depending on the sign of the size change on that branch. Consequently, the number of branches with expansions and contractions in the average profile pair is much greater using the gene tree based reconstruction (Figure 6). Moreover, after correcting for heterogeneous IC content, the sensitivity of gene tree based approaches is clearly greater than those using parsimony (Table 5).

## Conclusion

To summarize, our results confirm that the performance of phylogenetic profiling methods is limited in eukaryotic studies, an observation already made in previous studies of presence-absence profiling [9,10]. However, several methodological aspects beyond the scope of this article may influence the outcome: for example different methods for orthologous group prediction, large group decomposition and alternative gene tree building methods, different methods for species sampling or the use of other criteria to define *bona fide *functionally linked group pairs or alternative genetic event types that may also co-occur between co-evolving groups of genes.

In addition to presence-absence profiling, recent work in the field has proposed group size based approaches as an alternative methodology that may improve performance. Our analyses indicate that the relative performance of both approaches is for the most part dependent on the gene clustering granularity. While presence-absence profiling is more appropriate for fine-grained gene clusters such as the OrthoMCL orthologous groups, we show that the sensitivity of the group size based approaches can be increased by using coarser clusters of genes (based on the presence of a shared InterPro domain). In our experiments, the type of functional link affects both approaches in the same way, since direct protein-protein interactions (PPI) represent stronger interdependences and are generally better predicted than participation in the same KEGG pathway, which include a wider variety of relations. We also show that heterogeneous information content between positive and negative control datasets influences performance assessment. Moreover, a combination of two frequently adopted evaluation strategies resulted in a clear overestimation of the performance gained using group size approaches (i) generating a negative control set that involves random or exhaustive unweighted group pair generation and (ii) using a single null hypothesis rejection threshold. Here, we have proposed an alternative based on adjusting the profile pair scores based on their information content. In this context, weighted sampling of a negative set helps in obtaining enough cases across the information content range to accurately approximate this distribution with a smaller sampling.

In contrast with gene group size approaches, presence-absence profiling always shows a better positive versus negative classification accuracy in the face of the heterogeneous nature of the information content across profile pair sets. This property is true for both gene clustering granularities tested despite our efforts to improve gene group size based approaches using gene trees to infer ancestral group sizes. Therefore, we conclude that presence-absence profiling methods are more suitable for the analysis of datasets where the profiles have sufficient information content.

Nevertheless group size approaches are still useful and provide a complementary means of detecting domain or family level co-evolution between groups that may be elusive to presence-absence profiling approaches. Moreover positive correlation between co-evolution scores and functional links imply that these methods could be used to estimate functional distances between gene groups and to cluster them based on their functional relatedness.

Finally, despite the fact that this study focuses on eukaryotic genomes, we believe these observations should also be considered in future bacterial and archeobacterial phylogenetic profiling analyses.

## Authors' contributions

VRR performed all the required programming, data collection, analyses and drafted a first version of the manuscript. VRR and JDT jointly interpreted the results, modified and improved the manuscript. JDT and OP participated in the study design and management, and the revision of the manuscript. All authors read and approved the final manuscript.
